# Supplementary material for: Illegal Solid-Waste Dumping in a Low-Income Neighbourhood in South Africa: Prevalence and Perceptions
Source: Int J Environ Res Public Health. 2023 Sep 13;20(18):6750. doi: 10.3390/ijerph20186750 (PMC10530688; doi:10.3390/ijerph20186750)
Supplement: Supplementary file 1 [file ijerph-20-06750-s001.zip › ijerph-2396361-supplementary.pdf]

Supplementary Materials S1: HOUSEHOLD SURVEY QUESTIONNAIRE

Questionnaire no: ________ Date: _____________ Field worker name: ___________________________

Town: _________________________ Neighbourhood: Township [ ] RDP [ ]

Random sample point no._________________________

****

SECTION A: RESIDENTS' PRACTICES OF SOLID WASTE DUMPING

1.. How does your household dispose of municipal solid waste?

A. Open-land dumpsites
B. Burning of waste
C. Composting
D. Backyard disposal
E. Recycling
F. Landfill

2. Person(s) primarily responsible for disposing of waste in the household?

____

____

____

3. Frequency of disposal?

A. Daily
B. Weekly
C. Twice a week
D. Monthly
E. Twice Monthly
F. Other

4. Is there money spent on the disposal method? Yes [ ] No [ ]

If yes, how much is spent and for what?

____

____

____

5.Does the municipality offer waste-collection services in the area? Yes [ ] No [ ]

If yes, how frequent is this service?_____________________________

SECTION B: PERCEPTIONS ON THE DISTRIBUTION AND IMPACTS OF SOLID-WASTE DISPOSAL

12. What impact do poor SWD practices have on the surrounding environment?

____

____

____

13. What consequences does solid-waste dumping have on people? For example, health, livelihoods, livestock, and investments?

____
____
____

14. What kind of health consequences have you experienced or are aware of from the poor management of solid waste?

____
____
____

15. Who would you say is responsible for the disposal of solid waste?
   A. Municipal authorities
   B. Private companies
   C. Households
   D. NGOs
   E. Other

16. From your perspective, what factors contribute to illegal solid-waste dumpsites?

____
____
____

17. What could solve the problem of illegal solid-waste dumping?

____
____
____

18. Would you be open to learning about environmentally friendly SWD practices?
Yes [ ] No [ ]

SECTION C: RESPONDENT'S PROFILE

1. How long have you lived in this town? ____________________
2. What year were you born?____________________
3. What is your highest level of education? ____________________
4. What is your home language? isiXhosa [ ] isiZulu [ ] Afrikaans [ ] English [ ] Other [ ]
5. Gender? Female [ ] Male [ ] Non-binary [ ]
6. Number of people in the household?______________
7. What is your employment status? Employed [ ] Self-employed [ ] Unemployed [ ] Pensioned [ ]
8. What is the biggest source of your ***household*** income?
   Wages [ ] Social grants [ ] Private pensions [ ] Own business [ ]

9. Please indicate the broad range of the ***household*** monthly income (Rands)

<2,000 [ ] 2,001 – 6,000 [ ] 6,001 – 15,000 [ ] 15,0001 – 30,000 [ ] > 30,000 [ ]

10. Are you a member of any environmental society/agency/NGO? Yes [ ] No [ ]
